# Endocarditis due to *Abiotrophia defectiva*, a biofilm-related infection associated with the presence of fixed braces: A case report

**DOI:** 10.1097/MD.0000000000008756

**Published:** 2017-11-17

**Authors:** Victoria Birlutiu, Rares Mircea Birlutiu

**Affiliations:** a Faculty of Medicine, Lucian Blaga University of Sibiu, Sibiu; b Academic Emergency Hospital Sibiu—Infectious Diseases Clinic; c Spitalul Clinic de Ortopedie-Traumatologie si TBC osteoarticular “Foisor,” Bucuresti, Romania.

**Keywords:** *Abiotrophia defectiva*, biofilm, braces, infectious endocarditis

## Abstract

**Rationale:**

Endocarditis with Abiotrophia defectiva represents 4.3% to 6% of all streptococcal endocarditis. The article presents diagnosis issues and the complexity of the treatment.

**Patient concerns:**

We present the case of a female white patient, aged 26 years, who developed infectious endocarditis caused by A defectiva, in the last trimester of pregnancy, a biofilm-related infection associated with the presence of fixed braces.

**Diagnoses:**

The diagnosis of infectious endocarditis was confirmed by the cardiac ultrasound examination that revealed a voluminous vegetation on the mitral valve, and acute mitral regurgitation caused by chordae tendinae rupture, and also by isolating Abiotrophia defectiva from two positive blood cultures.

**Interventions:**

The decision to undergo surgical intervention was taken, and a mitral valve replacement was performed. Surgical intervention that was associated with board-spectrum antibiotic therapy.

**Outcomes:**

A defectiva, remains a rare cause of infective endocarditis, with a reserved prognosis that is motivated by the extensive valvular lesions and the risk of embolism.

**Lessons:**

The use of antibiotics administered in association, in the management of infective endocarditis, is mandatory.

## 1 Introduction

*Abiotrophia defectiva*, a nutritionally variant Streptococci (NVS) is found under normal conditions in the oral cavity, gastrointestinal tract, and genitourinary system. It has been involved in ocular infections, osteoarticular infections, prosthetic joint infections, otitis and sinus infections, cerebral abscesses, iatrogenic meningitis, and pancreatic abscesses. Endocarditis with *Abiotrophia* spp. represents about 4.3% to 6% of all streptococcal endocarditis. We present the first case of endocarditis with *A defectiva*, in a pregnant woman, associated with premature birth, and other various coinfections.

## 2 Case report

We present the case of a female white patient, aged 26 years, who denied any pathological diseases, except for repetitive episodes of pharyngitis. She was at the first pregnancy and was brought to the Obstetrics and Gynecology Department at 31 weeks of pregnancy for premature rupture of membranes and lumbosacral pain associated with mild left motor deficit, being suspected of a L5-S1 disc herniation. She delivered a 1550 g male fetus via caesarean section. She had had fixed braces for about a year, often causing gingival inflammation, for which her dentist recommended local antiseptics until delivery, further decisions being postponed. The postpartum clinical state was associated with frequent febrile episodes, apparently motivated by lactation. On the ninth day of postpartum confinement, she suddenly presented severe respiratory failure, an arterial oxygen saturation measured by pulse oximeter of 70% to 80% on simple face mask, dyspnea with tachypnea, disseminated crepitant rales in both lungs, heart rate of 180 to 190 beats/min, and marked alteration of her general condition.

The patient showed acute pulmonary edema, cardiogenic shock, requiring orotracheal intubation and mechanical ventilation. At the cardiovascular examination, a grade 3/6 systolic murmur, with left axillary irradiation was heard upon auscultation, which raised the suspicion of mitral valve injury—chordae tendineae rupture or infectious endocarditis.

Investigations were completed by a chest radiography that revealed prominent emphasized hilar interstitial drawing. Predominantly infrahilar, faint shaped, and bilateral parahilar micronodular, and subnodular opacities were also present. Bilateral, inferior lobe, and lung inhomogeneity was present being associated with clearing of the diaphragmatic perimeter.

A transesophageal echocardiography was also performed and revealed a massive mitral insufficiency with eccentric mitral regurgitation jets, and a 2-cm long, voluminous vegetation that was attached to the anteromiddle (A2) segment of the anterior mitral valve leaf (AMVL), with a ruptured chorda. Cauliflower-shaped hypermobile vegetative formations of 16-mm diameter were attached to the posteromedial papillary muscle (PM) of the left ventricle. At the level of the left atrium, hypoechogenic deposits of vegetation could be visualized. Also, the transesophageal echocardiography, revealed a severe pulmonary arterial hypertension with a pulmonary artery systolic pressure (PASP) of 60 + 20 mm Hg on the pulsed wave Doppler mode.

The decision to undergo surgical intervention was taken. A mitral valve replacement with a St Jude Medical no. 27 mechanical heart valve fixed with COR-KNOT device, through a minimal invasive approach—right mini-thoracotomy—was performed. The resumption of cardiac activity after declamping was in a third-degree atrioventricular block (AV block) with variable ventricular rate from the absence of ventricular depolarization to 50 to 60 beats/min, aspect which was maintained for 10 days, the reason why it was decided to implant a bicameral cardiac pacemaker in left subclavicular position.

From the harvested hemocultures and the bacteriological examination of the vegetation, *A defectiva* was identified using the VITEK 2 Compact analyzer (bioMérieux, Marcy-l’Étoile, France). Antibiotic sensitivity testing has not been performed. The most important laboratory studies are presented in Table 1.

**Table 1:**
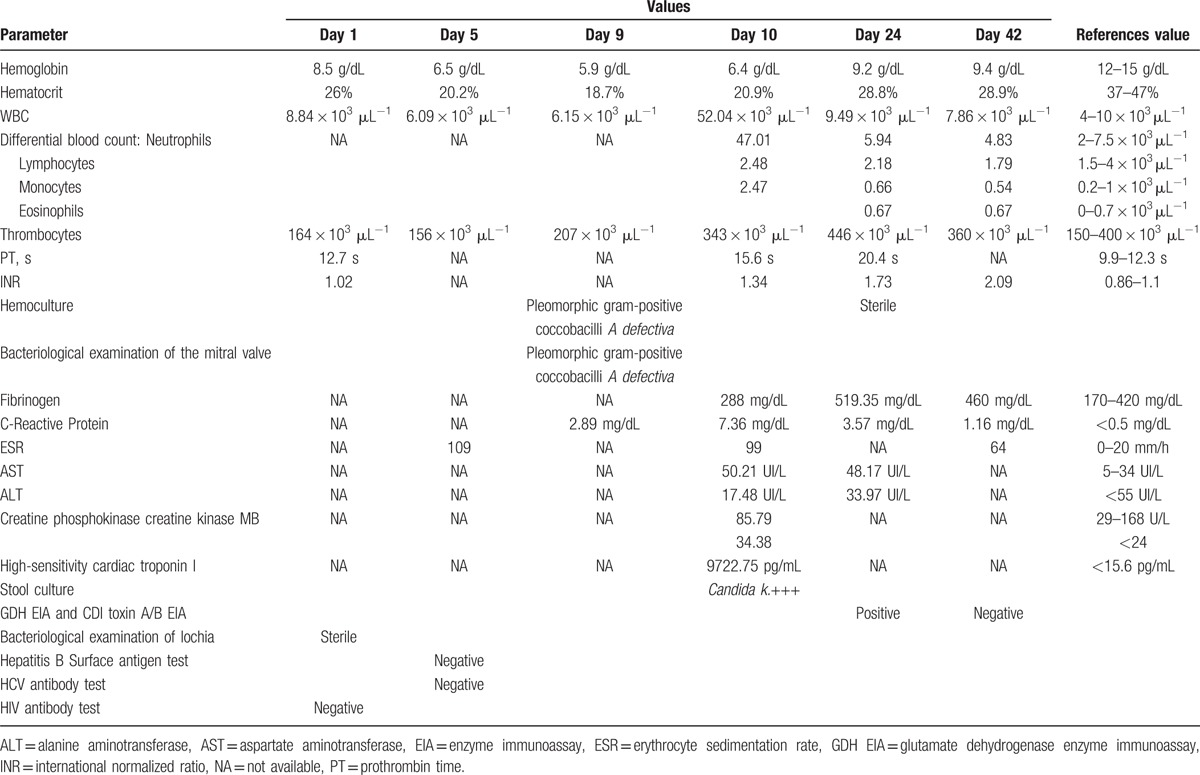
Laboratory studies.

The diagnosis of infectious endocarditis was confirmed by the cardiac ultrasound examination that revealed a voluminous vegetation on the mitral valve, and acute mitral regurgitation caused by chordae tendineae rupture, and also by isolating *A defectiva* from 2 positive blood cultures (2 samples drawn on the ninth day of hospitalization). Both major Duke criteria were met. Two of the minor criteria were also met (temperature >38.0°C (100.4° F and vascular phenomena).

Postoperatively and after resuming anamnesis the patient reported the presence of chills, fever not measured by thermometer, especially during night, fatigue, weight loss (BMI = 14.33 kg/m^2^) for about 1 month for which she did not present for medical consultation. During the third week of hospitalization, she had subungual bleeding at the left middle finger.

The evolution was slowly favorable under treatment with red blood cell transfusion, broad-spectrum antibiotics, meropenem 3 g/d (until final bacteriological examination, 6 days from the harvesting of the specimens) associated with vancomycin 2 g/d, gentamicin 160 mg/d, and caspofungin 50 mg/d. Since the seventh day of hospitalization, ceftriaxone 2 g/d plus gentamicin was continued for 2 weeks. After the surgical intervention, the respiratory evolution was rapidly favorable.

The occurrence of a diarrhea episode, confirmed to be related to a *Clostridium difficile* infection, required the discontinuation of cephalosporin therapy and the continuation with penicillin G 36 MU/d for another 3 weeks in parallel with oral vancomycin 4 × 125 mg/d for 14 days. The *C difficile* infection was diagnosed according to the guidelines published by the European Society of Clinical Microbiology and Infectious Diseases using 2 highly sensitive tests: GDH EIA (glutamate dehydrogenase enzyme immunoassay) and toxin A/B EIA. Both tests being positive and no further testing was required. Antibiotic therapy was associated with anti-clotting drugs, metoprolol 100 mg/d and perindopril 2 mg/d.

Prior to stopping penicillin treatment, it was decided to remove the fixed braces. The retrieved implants were introduced is a sterile container and saline sterile solution was added in this sterile container. The implants were processed within 30 minutes by sonication (1 minute) using an ultrasound bath (BactoSonic14.2, Bandelin GmbH, Berlin, Germany) at a frequency of 42 kHz and a power density of 0.22 W/cm^2^. The resulting sonication fluid is vortexed, and 50 mL of sonication fluid is centrifuged at 2500 rpm for 5 minutes. The resulting precipitate was inoculated onto Columbia agar with sheep blood (incubated aerobically, anaerobically, and in high concentration of CO_2_), Sabouraud plate, MacConkey agar plate, glucose broth, lactose broth, and thioglycollate broth. The cultures were incubated at 37°C for 14 days and inspected daily for bacterial growth. Isolated bacteria are identified using the VITEK 2 Compact analyzer (bioMérieux, Marcy-l’Étoile, France). The MICs (minimum inhibitory concentrations) are assessed according to the European Committee on Antimicrobial Susceptibility Testing breakpoints. After sonication, *Enterobacter cloacae* was identified with the following susceptibility. The strain was susceptible to ticarcillin, piperacillin, ceftazidime, aztreonam, meropenem, imipenem, amikacin, gentamicin, ciprofloxacin, and trimethoprim/sulfamethoxazole.

The echocardiographic follow-up on discharge was as follows nondilated left ventricle (45 mm), interventricular septum of 8 mm, posterior wall thickness of 9 mm, with preserved systolic function and an ejection fraction (EF) of 50%. Normokinetic, nondilated right ventricle of 31 mm. The echocardiography demonstrated the mechanical prosthesis with normal mobility for its elements as well as no periprosthetic regurgitation associated with maximum diastolic gradient estimated at 13 mm Hg (average gradient of 4 mm Hg) and mild aortic regurgitation as well as a mild tricuspid regurgitation. The systolic pulmonary artery pressure was of 20 + 10 mm Hg: 30 mm Hg, with an inferior vena cava of 16 mm, without inspiratory collapse in the context of mild hypervolemia. No pericardial effusion was revealed.

The favorable evolution of the patient was the result of an interdisciplinary collaboration between gynecologist, intensive care specialist, cardiovascular surgeon, cardiologist, infectious disease specialist, and microbiologist, who helped caring this case. The psychomotor development of the infant is according to his age.

## 3 Discussions

*A defectiva* was included in the genus *Abiotrophia*, along with *Abiotrophia adiacens*,^[1]^ from which it differs by the presence of alpha- and beta-galactosidases, respectively, by the absence of beta-glucuronidase. Subsequently, *A adiacens*, *A elegans*,^[2]^*A balaenopterae*,^[3]^ and *A para-adiacens*^[4]^ were reclassified as a new genus, *Granulicatella* gen. They are NVS, included in viridans streptococci, pleomorphic coccobacilli, non-motile, alpha-hemolytic colonies on blood agar, whose development on culture media requires complex enriched medium with vitamin B6 and l-cysteine. *A defectiva* possess virulence factors, such as the synthesis of exopolysaccharides, a component part of extracellular polymeric substances (EPSs), along with proteins and other constituents (lipids, DNA, etc), with a major role in biofilm formation,^[5–7]^ and moderate ability to bind to fibronectin. In vivo, the growth is supported by a variety of Gram-positive and Gram-negative bacteria. *A defectiva* is found under normal conditions in the oral cavity, gastrointestinal tract, and genitourinary system.^[8]^

*A defectiva* has been involved in ocular infections—endophthalmitis^[9]^ or keratitis,^[10]^ osteoarticular infections, arthroplasty infections,^[11–14]^ otitis and sinus infections, cerebral abscesses,^[15,16]^ iatrogenic meningitis, and pancreatic abscesses.^[17–19]^

Compared to beta-hemolytic streptococci (BHS), NVS have different susceptibility to antibiotics. NVS susceptibility to penicillin (8% according to some studies),^[20]^ carbapenems, cephalosporins, daptomycin, or macrolides is not certain.^[21,22]^

Endocarditis with *Abiotrophia* and *Granulicatella* represents about 4.3% to 6% of all streptococcal endocarditis,^[23]^ being recognized by complications such as acute heart failure, septic embolism, or extensive valvular lesions requiring surgical interventions in a larger percentage of cases compared with other non-hemolytic streptococci.^[24–34]^ Approximately 125 cases have been published in the literature so far of which in 1 published case the evolution has been complicated by hemophagocytic syndrome.^[22]^

The source of *A defectiva*, in our case, was the fixed braces, from which we identified, after 6 weeks of treatment, *Enterobacter cloaceae*, by sonication, a pathogen that possesses the ability to form biofilm on implants in association with other Gram-positive and Gram-negative bacteria. The particularity of our case lies in the fact that it is the first case of endocarditis with *A defectiva* in a pregnant woman, associated with premature birth, heart failure, pulmonary edema, and extensive valvular lesions requiring urgent surgical intervention. The postoperative evolution of the case has been associated with various coinfections—*C difficile* and *Candida krusei*—associations that required adjustments in the therapeutic management of the case in the absence of knowing the antibiotic susceptibility of the isolated strain of *A defectiva*.

The favorable evolution of our patient, under high doses of penicillin and gentamicin has shown that the isolated strain of *A defectiva* maintained its susceptibility to these antibiotics. We think that it would be wiser to perform a cardiac evaluation, especially in adults, at least for major oral cavity surgeries to exclude any possible cardiac lesion at risk of infectious endocarditis. Although we did not have confirmation of any previous heart disease in our patient's condition, poor nutritional status could have been the cause of an undiagnosed preexisting cardiac pathology.

### 3.1 Informed consent

Written informed consent was obtained from the patient for publication of this case report and any accompanying images. The study was accepted by the Ethics Committee of the hospital and they encouraged publishing the article. A copy of the written consent is available for review by the Editor-in-Chief of this journal.

## 4 Conclusion

*A defectiva* remains a rare cause of infective endocarditis, with a reserved prognosis that is motivated by the extensive valvular lesions and the risk for embolism. Almost 50% of the valvular lesions require surgical intervention of valve replacement. The variable susceptibility to antibiotics requires a close monitoring of the evolution of the cases. The use of antibiotics administered in association, in the management of infective endocarditis is mandatory.
